# The effect of NCMS on catastrophic health expenditure and impoverishment from tuberculosis care in China

**DOI:** 10.1186/s12939-016-0463-0

**Published:** 2016-10-18

**Authors:** Chengchao Zhou, Qian Long, Jiaying Chen, Li Xiang, Qiang Li, Shenglan Tang, Fei Huang, Qiang Sun, Henry Lucas, Shitong Huan

**Affiliations:** 1 Department of Social Medicine and Health Service Management, School of Public Health, Shandong University, Jinan, China; 2 Collaborative Innovation Center of Social Risks Governance in Health, School of Public Health, Shandong University, Jinan, China; 3 Key Laboratory of Health Economic and Policy Research, NHFPC, Shandong University, Wen-hua-xi Road No. 44, Jinan City, 250012 China; 4 Global Health Research Center, Duke Kunshan University, Kunshan, China; 5 School of Health Policy and Management, Nanjing Medical University, Nanjing, China; 6 School of Medicine and Health Management, Huazhong University of Science and Technology, Wuhan, China; 7 School of Public Health, Xi’an Jiaotong University, Xi’an, China; 8 Duke Global Health Institute, Duke University, Durham, NC USA; 9 National Center for TB Control and Prevention, China CDC, Beijing, China; 10 Center for Health Management and Policy, Shandong University, Jinan, China; 11 Institute of Development Studies, Sussex University, Brighton, UK; 12 Bill & Melinda Gates Foundation Beijing Office, Beijing, China

**Keywords:** NCMS, Catastrophic health expenditure, Impoverishment, Tuberculosis, China

## Abstract

**Background:**

Health expenditure for tuberculosis (TB) care often pushes households into catastrophe and poverty. New Cooperative Medical Scheme (NCMS) aims to protect households from catastrophic health expenditure (CHE) and impoverishment in rural China. This article assesses the effect of NCMS on relieving CHE and impoverishment from TB care in rural China.

**Methods:**

Three hundred fourty-seven TB cases are included in the analysis. We analyze the incidence and intensity of CHE and poverty, and assess the protective effect of NCMS by comparing the CHE and impoverishment before and after reimbursement.

**Results:**

After out-of-pocket (OOP) payment for TB care, 16.1 % of non-poor fall below poverty line. The NCMS reduces the incidence of CHE and impoverishment by 11.5 % and 7.3 %. After reimbursement, 46.7 % of the households still experience CHE and 35.4 % are below the poverty line. The NCMS relieves the mean gap, mean positive gap, poverty gap and normalized positive gap by 44.5 %, 51.0 %, US$115.8 and 31.6 % respectively.

**Conclusions:**

The NCMS has partial effect on protecting households from CHE and impoverishment from TB care. The limited protection could be enhanced by redesigning benefit coverage to improve the “height” of the NCMS and representing fee-for-service with alternative payment mechanisms.

**Electronic supplementary material:**

The online version of this article (doi:10.1186/s12939-016-0463-0) contains supplementary material, which is available to authorized users.

## Background

China ranks second on the list of countries with the largest burden of tuberculosis (TB) in the world [1]. TB is often regarded as “a disease of the poor” [2, 3]. There is an obvious vicious circle between TB and poverty. On the one hand, the poor are at a high risk of TB infection and have higher TB prevalence and incidence [3–7]. On the other hand, TB contributes much to poverty by affecting negatively the labor supply which would result in a severe loss of future earning for patients’ families [3, 8–10]. Furthermore, many studies have documented that TB patients incur high costs for diagnosis and treatment despite TB care offered for free in most settings, and households often have to borrow money or sell assets to pay for TB care, which places the patients’ households at higher risk of catastrophic health expenditure (CHE) and pushes them into poverty [11–13].

Health insurance has been seen as an effective way to reduce economic burdens of illness and protect households from CHE and impoverishment [14–16]. In China, health insurance system consists of three separate schemes, among which New Cooperative Medical Scheme (NCMS) is most an important scheme for rural residents. The NCMS, started in 2003, is a voluntary program based on a joint funding from the central, provincial, county level governments and also enrollees premium [17–20]. The central government is responsible for the scheme management and supervision, while the scheme implementation responsibilities are decentralized to local governments. By the end of 2011, NCMS had covered about 832 million rural population in China, and the coverage had increased to 97.5 %. Meanwhile, the NCMS revenues per capita increased to 250 RMB (39.8 US$)^1^ in 2011 [21].

The primary objective of the Chinese health insurance schemes is to prevent the population from impoverishing for health expenditures. Some studies have documented that health insurance can protect patients from CHE, although in some countries the effect is partial or limited [15, 22–24]. There is, however, no studies reporting the health insurance effect on CHE and impoverishment for TB care. To evaluate the health insurance impact on catastrophic health expenditure and impoverishment for TB, a disease strongly associated with poverty, is of high priority. This study aims to analyze the incidence and intensity of CHE and impoverishment for TB care, assess the effect of NCMS, and also governmental and other subsidies on CHE and impoverishment, so as to provide policy implications for NCMS and governmental subsidy in preventing CHE and impoverishment from TB care.

## Methods

### Study participants

The present study uses the data of the baseline survey of the Gates Foundation Phase II program. The baseline survey is conducted in Zhenjiang City of Jiangsu Province; Yichang City of Hubei Province and Hanzhong City of Shaanxi Province. Three counties or districts (one from each category of high, middle and low GDP per capita) are selected as study sites in each city (Dantu, Yangzhong and Jurong in Zhenjiang; Zhijiang, Yidu and Wufeng in Yichang; and Chenggu, Mian and Zhenba in Hanzhong). The NCMS coverage rates in all of the study sites were over 95 % by the end of 2011, which were similar with that at the national level. The NCMS offices of the study sites are responsible for qualifying who and how much the NCMS would cover.

In the selected study sites, TB cases are diagnosed and treated in local TB dispensaries (LTD) or designated hospitals, which are also similar with that elsewhere. Those lower levels of providers, such as township health centers and clinics, cannot diagnose and treat the TB cases, but only to detect the cases as suspicious patients and then referred to the LTD or designated hospitals. All diagnosed TB cases (including new and relapsed patients) are required to be registered in the local dispensaries and reported to upper level health authorities. The participants of this study are selected by using a two-stage cluster random sampling method. In the first stage, three townships/streets are randomly selected in each sampling county/district. In the second stage, about 30 TB cases are randomly selected from the list of registered cases in each township/street. The participants could be new cases or retreated cases, but should meet the criteria that they had completed the normal treatment or stop treatment during 2012. A total of 347 cases with complete data and covered by the NCMS are included in the analysis.

### Data collection

A standardized questionnaire is used for the survey. The questionnaire includes a detailed list of the total expenses before reimbursement, the NCMS reimbursement for TB care, out-of-pocket (OOP), medical and non-medical costs incurred in the pathways to TB diagnosis and treatment, household income, household expenditure, subsidies from government and others. Furthermore, demographic and socio-economic information of patients and their households are also included, such as age, sex, education, and marital status.

In this study, OOP payments and total health expenditure on TB care were collected by the TB patients per individual. If the family has more than one TB patients, we will obtain the sum of OOP payments and health expenditure on TB care of all TB patients in the same family as a whole. The household expenditure, food expenditure, and capacity to pay were collected by houses, not individuals.

The patient survey was conducted by university/college students from Huazhong University of Science and Technology (Yichang), Xi’an Jiaotong University (Hanzhong) and Nanjing Medical University (Zhenjiang). The technical assistance team (TA) from Duke Global Health Institute, US, Sussex University, UK, and Shandong University, China also took part in the baseline survey. The interviewers had received an appropriate training on interview skills and the contents of the questionnaire before the survey. All the patients were interviewed in local TB dispensaries, designated hospitals or in local township health centers during April to May in 2013.

### Definitions

CHE is usually assessed by incidence and intensity. Head count (HC) is used to measure incidence, mean gap (MG) and mean positive gap (MPG) are used to reflect intensity. HC means the percentage of households whose OOP payments as a proportion of income, equals or exceeds a threshold. There is no consensus on the thresholds, but the two most commonly used are: 10 % of total household income, and 40 % of household capacity to pay. In this study, we empirically use the latter to measure CHE. That is to say, the total OOP payments equaling or exceeding 40 % of the household capacity to pay is regarded as catastrophic. HC is estimated as follows:

$$ HC=\frac{1}{\mathrm{N}}{\displaystyle \sum_{i=1}^N}E $$

Where N is the sample size, E is an indicator equal to 1 if OOP of a household *i* as a proportion of its capacity to pay is greater than the threshold *Z* and zero otherwise. MG is the average amount by which payments, as a proportion of income, equal or exceed 40 % of their non-food income. It is estimated as follows:

$$ MG=\frac{1}{\mathrm{N}}{\displaystyle \sum_{i=1}^N}G $$

Where N is the sample size, G is an indication of how much OOP exceed the threshold, equal to T_*i*_/X_*i-*_ *Z* if T_*i*_/X > *Z* and zero otherwise. Here, T*i* is the OOP payments of household *i*, X*i* is the household capacity to pay and *Z* is the threshold share. MPG is defined as the head count being a fraction of the MG.

The national poverty line of Chinese RMB 2300 Yuan (366.2 US$) per person per year (equal to international poverty line of US$ 1 Yuan per day, a currency exchange rate of Chinese RMB 628 Yuan to US$100 Yuan at the end of 2012) is used to estimate poverty levels before and after reimbursement. This poverty line was defined by China government in 2011, and could provide a criterion for comparisons before and after NCMS reimbursement for TB care. Similar to the measurement of CHE, four indicators are presented to measure poverty. Similar with CHE, poverty is also assessed by incidence and intensity. Poverty head count is used to measure incidence, poverty gap (PG), normalized poverty gap (NPG), and mean positive poverty gap (MPPG) are used to reflect intensity. Poverty head count means the proportion of households living below the poverty line. PG is defined as average of all shortfalls from the poverty line. NPG is obtained by dividing the poverty gap by the poverty line. MPPG is defined as the poverty head count being a fraction of the poverty gap [25, 26]. The estimation methods of the four indicators of poverty are similar with those of CHE. Adam Wagstaff and Eddy van Doorslaer have reported the methodology in detail [26].

OOP payments for TB care in this study include direct health expenditures on diagnosis and treatment (consultation fees, laboratory tests, X-rays, drugs, and hospital care), associated transport and accommodation costs for patients and companions, nutrition supplements cost and others. Insurance costs are not included in the OOP payments and health expenditures.

Social demographic characteristics include gender, age, type of TB cases, education, and marital status. The age of the participants is grouped into three types: ≤40, 41–59 and ≥60 years. Other demographic characteristics are categorized as follows: gender (male vs. female), type of TB cases (new vs. relapse), education (none, primary school, junior school and senior school or above), and marital status (married, single and bereft of spouse).

### Data analysis

The data is double entered and checked using EPI Data 6.04. SPSS 13.0 is used to analyze the data. Expenses including total expenditure on TB care, OOP, household income, and capacity to pay are presented as means and standard deviation. Head count is described as proportion. To evaluate the effect of NCMS and governmental subsidies on CHE and impoverishment, three scenarios are considered: (i) before reimbursement; (ii) after reimbursement from NCMS; and (iii) after reimbursement from NCMS and subsidies from the government and other sources. The difference is obtained between before and after reimbursement from NCMS, and also subsidies.

## Results

The mean age is 54.1 years (SD = 14.4 years) with a median of 57.0 years (15 to 86 years). Of the participants, 74.9 % males and 25.1 % females; 19.0 % never educated, 32.6 % primary education, 36.9 % junior education, and 11.5 % senior education and above. With regard to the patients, 83.0 % are new cases or never treated, while 17.0 % are relapsed or previously treated. As for marital status, 81.8 % married, 4.6 % single, and 13.5 % bereft of spouse (see Table 1).

**Table 1:** Socio-demographic characteristics of the participants

Characteristic	Patients No.	Percent
Observations	347	100.0
Sites		
Zhenjiang	39	11.2
Yichang	130	37.5
Hanzhong	178	51.3
Gender		
Male	260	74.9
Female	87	25.1
Age (years)		
≤40	57	16.4
41–59	148	42.7
≥60	142	40.9
Type of TB case		
New	288	83.0
Relapse	59	17.0
Education		
None	66	19.0
Primary school	113	32.6
Junior school	128	36.9
Senior school or above	40	11.5
Marital Status		
Married	284	81.8
Single	16	4.6
Bereft of spouse	47	13.5

The direct health expenditure for TB care is the most important component of the OOP, accounting for over 72 % of the total OOP payments, followed by transport and accommodation costs for patients and companions (13.3 %), and nutrition supplements cost (13.1 %). We presented the details of the components of OOP payments in Additional file 1: Table S1, and interested readers are encouraged to refer to that table.

Table 2 shows that 58.2 % of patients experience CHE before reimbursement for TB care. After reimbursement, the CHE head count falls to 46.7 %. When taking the subsidies into account, we find that there is a further decrease of head count in Zhenjiang (2.6 %) and Hanzhong (0.6 %) after subsidies from government and other sources. Table 3 shows the NCMS effect on intensity of CHE. Before reimbursement, the MG and MPG are 97.4 and 186.8 %. After reimbursement, the MG and MPG fall to 52.9 and 135.8 % respectively and further to 49.8 and 130.0 % after subsidies.

**Table 2:** NCMS^a^ impact on incidence of catastrophic expenditure for TB care, China, 2012

Indicators	Study locations			All
	Hanzhong	Yichang	Zhenjiang	
Average capacity to pay (US$) (A1)	2436.6 (1781.0)^b^	3242.8 (2245.4)	5143.7 (3219.3)	3042.9 (2268.5)
Average food expenditure (US$) (A2)	958.0 (444.8)	1157.5 (501.9)	1906.7 (708.4)	1139.4 (530.1)
Ratios of A2 versus A1	1 : 2.54	1 : 2.80	1 : 2.70	1 : 2.67
Total expenditure on TB care (US$^c^)	1696.6 (1201.1)	1257.7 (911.4)	2226.9 (904.4)	1591.8 (1074.4)
OOP^d^ payments for TB care (US$)	1094.1 (900.5)	898.9 (736.5)	1592.8 (649.5)	1077.0 (817.4)
OOP payments share of total expenditure on TB care (%)	64.5	71.5	71.5	67.7
Reimbursement from health insurance system (US$)	589.2	304.6	619.0	485.9
NCMS share of total expenditure on TB care (%)	34.7	24.2	27.8	30.5
Subsidies from government and other sources (US$)	13.4	54.25	15.1	28.9
Subsidy share of total expenditure on TB care (%)	0.8	4.3	0.7	1.8
Households with catastrophic expenditure (%)				
*Before reimbursement (C*_*B*_ *)*	63.5	50.8	59.0	58.2
*After reimbursement (C*_*A*_ *)*	51.1	39.2	51.3	46.7
*After reimbursement and subsidies (C*_*S*_ *)*	50.6	39.2	48.7	46.1
Difference (%)				
*C*_*B*_ *-C*_*A*_	12.4	11.6	7.7	11.5
*C*_*A*_ *-C*_*S*_	0.6	0.0	2.6	0.6
*C*_*B*_ *-C*_*A*_ *-C*_*S*_	13.0	11.6	10.3	12.1

^a^NCMS means New Cooperative Medical Scheme, the same below;

^b^Mean (SD)

^c^A currency exchange rate of Chinese RMB 628 Yuan to US$1 00 Yuan (at the end of 2012);

^d^OOP : out-of-pocket

**Table 3:** NCMS impact on intensity of catastrophic expenditure for TB care, China, 2012

Indicators	Study locations			All
	Hanzhong	Yichang	Zhenjiang	
Mean Catastrophic Payment Gap (%)				
*Before reimbursement (MG*_*B*_ *)*	155.2	51.7	85.4	97.4
*After reimbursement (MG*_*A*_ *)*	68.6	35.5	43.0	52.9
*After reimbursement and subsidies (MG*_*S*_ *)*	65.6	31.3	42.7	49.8
Mean positive gap (%)				
*Before reimbursement (MPG*_*B*_ *)*	237.7	115.4	153.7	186.8
*After reimbursement (MPG*_*A*_ *)*	161.8	105.6	91.0	135.8
*After reimbursement and subsidies (MPG*_*S*_ *)*	157.3	98.2	90.5	130.0
Difference (%)				
*MG*_*B*_ *-MG*_*A*_	86.6	16.2	42.4	44.5
*MG*_*A*_ *-MG*_*S*_	3.0	4.2	0.3	3.1
*MPG*_*B*_ *-MPG*_*A*_	75.9	9.8	62.7	51.0
*MPG*_*A*_ *-MPG*_*S*_	4.5	7.4	0.5	5.8

Without considering OOP payments for TB care, poor households (below the poverty line) account for 19.3 % of all households and over 97.0 % of the households are in the poorest quintile (Q1). After OOP payments for TB care, we find 16.1 % of non-poor households become poor. Impoverishment from TB care is more common in the poorer quintiles (Q1, Q2) (Table 4).

**Table 4:** Headcounts of prepayment poverty and of poverty impoverished by TB OOP expenses, China, 2012

Characteristic	H_pre_ (%)	H_oop_ (%)	Impoverished (%)
Study locations			
*Hanzhong*	26.4	44.4	18.0
*Yichang*	12.3	26.2	13.1
*Zhenjiang*	10.3	25.6	15.3
Household income quintiles^a^			
*Q1*	60.6	84.4	13.8
*Q2*	1.8	29.8	28.0
*Q3*	0.0	12.3	12.3
*Q4*	0.0	4.0	4.0
Total	19.3	35.4	16.1

^a^Quartile1 (Q1) is the poorest and Quartile 4 (Q4) is the richest

Before reimbursement, households below poverty line account for 42.7 %. The NCMS reduces the headcount rate by 7.3 % and the before and after difference is highest in Hanzhong (9.0 %). But the subsidies have no effect on decreasing impoverishment (Table 5). Table 6 shows the NCMS on intensity of impoverishment. The NCMS decreases the PG, NG and MPPG by 115.8 US$, 31.6 %, and 183.3 US$ respectively. However, the subsidies only have a very small impact on the three above-mentioned indicators, which are 7.4 US$, 2.0 %, and 20.9 US$ respectively.

**Table 5:** NCMS impact on incidence of impoverishment for TB care, China, 2012

Indicators	Study locations			All
	Hanzhong	Yichang	Zhenjiang	
Poverty line (US$^a^)	366.2	366.2	366.2	366.2
Poverty headcounts (%)				
*Before reimbursement (P*_*B*_ *)*	53.4	31.5	30.8	42.7
*After reimbursement (P*_*A*_ *)*	44.4	26.2	25.6	35.4
*After reimbursement and subsidies (P*_*S*_ *)*	44.4	26.2	25.6	35.4
Difference (%)				
*P*_*B*_ *-P*_*A*_	9.0	5.3	5.2	7.3
*P*_*A*_ *-P*_*S*_	0.0	0.0	0.0	0.0

^a^A currency exchange rate of Chinese RMB 628 Yuan to US$1 00 Yuan (at the end of 2012)

**Table 6:** NCMS impact on intensity of impoverishment for TB care, China, 2012

Indicators	Study locations			All
	Hanzhong	Yichang	Zhenjiang	
Poverty gap (US$^a^)				
*Pre-payment (PG*_*pre*_ *)*	45.2	14.0	8.6	26.0
*Before reimbursement (PG*_*B*_ *)*	465.6	188.8	237.4	297.7
*After reimbursement (PG*_*A*_ *)*	285.9	118.9	126.7	181.9
*After reimbursement and subsidies (PG*_*S*_ *)*	280.1	106.6	120.6	174.5
*Difference* (US$)				
*PG*_*B*_ *-PG*_*A*_	179.7	69.9	110.7	115.8
*PG*_*A*_ *-PG*_*S*_	5.8	12.3	6.1	7.4
*PG*_*B*_ *-PG*_*pre*_	420.4	164.8	228.8	271.7
*PG*_*S*_ *-PG*_*pre*_	234.9	92.6	112.0	148.5
Normalized poverty gap (%)				
*Pre-payment (NPG*_*pre*_ *)*	12.3	3.8	2.3	7.1
*Before reimbursement (NPG*_*B*_ *)*	127.1	51.6	64.8	81.3
*After reimbursement (NPG*_*A*_ *)*	78.1	32.5	34.6	49.7
*After reimbursement and subsidies (NPG*_*S*_ *)*	76.5	29.1	32.9	47.7
*Difference* (%)				
*NPG*_*B*_ *-NPG*_*A*_	49.1	19.1	30.2	31.6
*NPG*_*A*_ *-NPG*_*S*_	1.6	3.4	1.7	2.0
*NPG*_*B*_ *-NPG*_*pre*_	114.8	47.7	62.5	74.2
*NPG*_*S*_ *-NPG*_*pre*_	64.1	25.3	30.6	40.6
Mean positive poverty gap (US$)				
*Pre-payment (MPPG*_*pre*_ *)*	171.2	113.8	83.5	134.7
*Before reimbursement (MPPG*_*B*_ *)*	871.9	599.4	770.8	697.2
*After reimbursement (MPPG*_*A*_ *)*	643.9	453.8	494.9	513.8
*After reimbursement and subsidies (MPPG*_*S*_ *)*	630.9	406.9	471.1	492.9
*Difference* (US$)				
*MPPG*_*B*_ *-MPPG*_*A*_	228.0	145.5	275.9	183.3
*MPPG*_*A*_ *-MPPG*_*S*_	13.1	46.9	23.8	20.9
*MPPG*_*B*_ *-MPPG*_*pre*_	700.7	485.5	687.3	562.5
*MPPG*_*S*_ *-MPPG*_*pre*_	459.6	293.0	387.6	358.2

^a^A currency exchange rate of Chinese RMB 628 Yuan to US$1 00 Yuan (at the end of 2012)

## Discussion

The participants of this study are NCMS enrollees. Those who are not covered by the scheme or who cannot afford the deductible are not included in our study, which would probably lead to an underestimation of incidence and intensity of CHE and impoverishment. We collect the data mainly by self-reported investigations. Data on household income, food consumption, expenses on TB care and NCMS claims are probably inaccurate for interviewees’ recall bias or their unwillingness to tell the truth. Due to the limitations of cross-sectional study design, we mainly use some descriptive indicators to evaluate the effect, and we cannot interpret the findings as cause and effect. In addition, the sample size of this study is a little small, which might affect the statistical power to some extent. Despite these limitations, the findings of this study would offer an insight into the effect of NCMS and governmental subsidy on protection from CHE and impoverishment for TB care.

We find that TB places a really heavy financial burden on those sufferings. The health expenditure for TB care before reimbursement accounts, in average, for 52.3 % of the annual household capacity to pay. It is much higher than what is reported for chronic disease in Shandong (27 %) and Ningxia (35 %) [22]. The proportion of households whose total expenditure on TB care equals or exceeds 40 % of non-food income is 58.2 % before reimbursement, which is also much higher than the reported for chronic disease in Shandong and Ningxia (varied from 14–21 %). The OOP payments for TB care account for about an average of 35.4 % of the annual household non-food income, which is a little higher than that for TB care in Nigeria (29.1 %) [27], and also much higher than those reported in other countries [16].

The study shows that TB has a significant impoverishing effect in rural China. A study of 11 Asian countries survey showed that the OOP payments for healthcare raised the poverty head count by 2.7 % at the international line [28]. In China, a study conducted in 1998 found that OOP payments for healthcare added the rate from 3.26 to 7.22 % at the Chinese official poverty line [29]. A study using the data from 2000 Chinese National Bureau of Statistics showed that OOP payments for healthcare increased the poverty rate by 2.6 % at the poverty line of US $1.08 per day [28]. Another study using the data of the China Fourth National Health Service Survey (2008) indicated that the OOP payments pushed 7.0 % of non-poor into the poor at the poverty line of US$ 449.40 per year [30]. The impoverishment from OOP for TB care of our study (16.1 %) is much higher than that of the above studies. Similar to previous study, we also find that impoverishment is more common in poorer TB patients [30].

Our findings show that the NCMS provide some financial protection for TB patients. We also find that there is a certain reduction in intensity indicators including MG, MPG, PG, NPG and MPPG. This finding is consistent with other studies conducted in Anhui, Chongqing, Qinghai, Shandong and Ningxia of China, and in Gujarat of India for health expenditure on other diseases [22, 23, 31].

However, this protection is only limited and the expenses for TB care after reimbursement still pose a threat to some households. Our study finds that 46.7 % of the households still experience CHE and 35.4 % are still below the poverty line even after reimbursement. It implies that there is a big gap between actual protection level and the ideal goal of the policy design for the NCMS. Over the last decade, China has made great strides in NCMS coverage [21]. But we still need to try to achieve the goal of universal coverage. The universal coverage policy declared by WHO includes three dimensions: breadth, depth and height [30, 32]. In this study, the ‘height of coverage’ of NCMS, which refers to the extent to which health service costs are covered, is relatively low and insufficient to reach the goal of the universal coverage. This poor ‘height of coverage’ of NCMS for TB care is mainly due to two aspects of reasons. One is the potential shortcomings of the policy design for NCMS, and the other is the high cost for TB care.

In China, the government commits to provide free diagnosis and treatment for TB cases. However, the providers, under a circumstance of FFS system, still have strong incentives to prescribe expensive medical examinations and new drugs beyond the free service package recommended by the national TB program [30, 33]. Previous studies indicated that the doctors recommend liver protection and ancillary drugs, follow-up X-rays even CT tests and blood tests which would add to OOP payments [11, 34, 35]. This supplier-induced demand on a FFS basis has contributed to a rapid increase in TB care costs. In order to effectively contain costs on TB-related healthcare, this project will carry out an intervention of case-based payment model in the further study, and will examine the impact of the new payment model on CHE and impoverishment.

Our results indicate that subsidies from the government and other sources (mainly refers to Medical Financial Assistance, MFA) have little effect on the protection against CHE and impoverishment. There are two explanations for the relatively little effect in our study. First, the amount of subsidies from the government and other sources are relatively small. When compared with the large amount of total expenditure for TB care, the effect of subsidies on financial protection is greatly undermined. Second, the limited financial assistance fund might be used mainly to increase the “breadth” rather than to improve the “height”. Therefore, TB control program should address the medical subsidies to maximize its efficiency by expanding the subsidy sources and assisting the most really vulnerable patients so as to improve the “height”.

## Conclusion

This study finds that NCMS has partial effect on protecting households from CHE and impoverishment from TB care, and governmental and other subsidies have little effect on this protection. Our findings present four important implications for health policymakers. First, TB places a really heavy financial burden on households suffered from TB in China. It is vital to develop TB-targeting policies to protect against financial risk, such as establishing special fund for TB or bringing TB (for those poor sufferings) into the package of the most serious diseases affecting Chinese population. Second, the impoverishment from TB care is more common in poorer households. There is a need to develop pro-poor interventions. A mix of health insurance schemes and MFA for the poor might be useful for providing a financial protection. Third, the NCMS aims to protect enrollees against CHE and impoverishment, but this aim is only partly achieved due to the high costs for TB care and poor ‘height of coverage’. It suggests for decision makers to develop comprehensive strategies to address the problem. This might be achieved through redesigning benefit coverage to improve the “height” of the NCMS, representing FFS with alternative payment mechanisms such as case-based payment and extending broadly the TB free service package. Finally, the subsidies from government and other sources have little effect on financial protection against CHE and impoverishment. It implicates for policymakers to improve the subsidy height by assisting those who were really catastrophic.

## Additional file

Additional file 1: Table S1. Main components of OOP^a^ payment for TB care in China, 2012. **Table S2.** Demographic profile of the study sites in 2012, China. (DOCX 16 kb)

## Abbreviations

CHE: Catastrophic health expenditure
FFS: Fee-for-service
HC: Head count
MFA: Medical financial assistance
MG: Mean gap
MPG: Mean positive gap
MPPG: Mean positive poverty gap
NCMS: New cooperative medical scheme
NPG: Normalized poverty gap
OOP: Out-of-pocket
PG: Poverty gap

## Footnotes

1 A currency exchange rate of Chinese RMB 6.28 Yuan to US$1 Yuan (at the end of 2012), and it is the same below.
