# Supplementary material for: The effect of NCMS on catastrophic health expenditure and impoverishment from tuberculosis care in China
Source: Int J Equity Health. 2016 Oct 18;15:172. doi: 10.1186/s12939-016-0463-0 (PMC5069881; doi:10.1186/s12939-016-0463-0)
Supplement: Additional file 1: Table S1. — Main components of OOPa payment for TB care in China, 2012. Table S2. Demographic profile of the study sites in 2012, China. (DOCX 16 kb) [file 12939_2016_463_MOESM1_ESM.docx]

Additional file

Table S1. Main components of OOP^a^ payment for TB care in China, 2012

| **Main components of OOP payments ^b^(US$^c^)** | **Hanzhong** | **Yichang** | **Zhenjiang** | **ALL** |
| --- | --- | --- | --- | --- |
| Direct health expenditure | 761.0 ( 653.5)^d^ | 706.2 (673.4) | 1114.3 (492.4) | 780.1 (643.4) |
| Transport and accommodation costs | 177.7 (136.2) | 104.9 (104.4) | 116.9 (76.2) | 143.6 (119.2) |
| Nutrition supplements cost | 151.5 (52.0) | 83.2 (53.6) | 291.1 (39.2) | 141.6 (49.2) |
| Total | 1094.1 (900.5) | 898.9 (736.5) | 1592.8 (649.5) | 1077.0 (817.4) |

a. OOP : Out-of-pocket;

b. Components of OOP payments including: direct health expenditure, transport and accommodation costs, nutrition supplement cost and others (i.e., payment for someone who

take care of you);

c. A currency exchange rate of Chinese RMB 628 Yuan to US$1 00 Yuan (at the end of 2012);

d. Mean (SD)

Table S2. Demographic profile of the study sites in 2012, China

| **Study sites** | **Population size**  **(thousand)** | **GDP^a^ per capita (US$^b^)** | **Provincial GDP per capita (US$)** |
| --- | --- | --- | --- |
| Hanzhong | 342,6 | 2,697 | 6,140 |
| Yichang | 4,088 | 8,959 | 6,142 |
| Zhenjinag | 3,155 | 11,775 | 10,883 |

a. GDP: Gross domestic products;

b. A currency exchange rate of Chinese RMB 628 Yuan to US$1 00 Yuan (at the end of 2012).,
